# Hemodynamic Implications of Prone Positioning in Patients with ARDS

**DOI:** 10.1186/s13054-023-04369-x

**Published:** 2023-03-21

**Authors:** Christopher Lai, Xavier Monnet, Jean-Louis Teboul

**Affiliations:** AP-HP, Service de Médecine Intensive-Réanimation, Hôpital de Bicêtre, DMU CORREVE, Inserm UMR S_999, FHU SEPSIS, Groupe de Recherche Clinique CARMAS, Université Paris-Saclay, Le Kremlin-Bicêtre, France

## Abstract

This article is one of ten reviews selected from the Annual Update in Intensive Care and Emergency Medicine 2023. Other selected articles can be found online at https://www.biomedcentral.com/collections/annualupdate2023. Further information about the Annual Update in Intensive Care and Emergency Medicine is available from https://link.springer.com/bookseries/8901.

## Introduction

Prone positioning is recommended in patients with moderate to severe acute respiratory distress syndrome (ARDS) when the ratio of arterial oxygen partial pressure (PaO_2_) to inspired oxygen fraction (FiO_2_) is < 150 mmHg despite optimized mechanical ventilation or use of neuromuscular blockade [1, 2]. Indeed, prone position may improve arterial oxygenation [3, 4] and sessions lasting more than 16 h are associated with reduced mortality [5]. The use of prone positioning has spread considerably in recent years. Whereas in a large observational study in 2014 only 16% of patients with severe ARDS underwent prone positioning [6], more than two thirds of patients with moderate-to-severe ARDS had several sessions of prone positioning during the first wave of the coronavirus disease 2019 (COVID-19) pandemic in 2020 [7, 8].

Prone positioning improves oxygenation through improvement of the ventilation-to-perfusion ratio since aeration and ventilation increase in the most dorsal parts of the lung, whereas pulmonary blood flow remains predominant in these parts [9]. Lung recruitment also permits a decrease in atelectrauma, reduction in the transpulmonary driving pressure, and increase in lung compliance [10]. Hence, prone positioning may limit the mechanical power [11] and might thus prevent ventilator-induced lung injury (VILI).

In addition to the effects on oxygenation and respiratory mechanics, prone positioning induces some hemodynamic effects, which may also be beneficial [12, 13]. In this article, we review how prone positioning can exert those favorable cardiovascular effects. Moreover, prone position sessions are at least 16 h long [1], and even sometimes extended up to 39 h [14]. During such a long time period, the question of administering fluid therapy may arise. Thus, we will explore how preload responsiveness could be detected to guide fluid therapy in patients in the prone position.

## Hemodynamic Effects of Prone Positioning

### Prone Positioning Affects Venous Return Determinants and May Increase Right Ventricular Preload

**Fig. 1:**
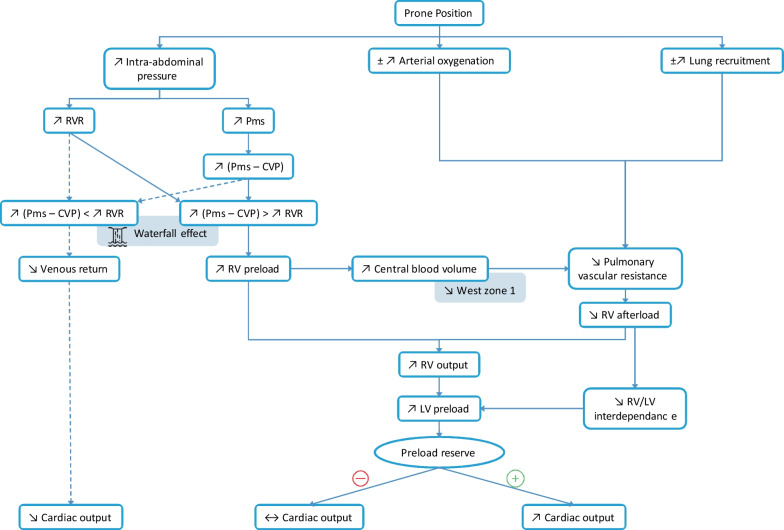
Hemodynamic effects of prone positioning. *CVP* central venous pressure, *LV* left ven- tricular, *Pms* mean systemic pressure, *RV* right ventricular, *RVR* resistance to venous return

Venous return is the blood flow from the systemic venous network towards the right heart [15]. According to Guyton’s model, venous return is equal to the venous return pressure gradient divided by the resistance to venous return [16]. The pressure gradient of venous return is defined as the difference between the mean systemic pressure (Pms) and the right atrial pressure [16]. Prone positioning increases the intra-abdominal pressure (IAP) [12, 17] (Fig. 1). This may cause two distinct effects on venous return. On the one hand, prone positioning increases Pms and central venous pressure (CVP) to a lesser extent, resulting in an increase in the pressure gradient of venous return [17] (Fig. 1). These effects are due: (1) to lowering the trunk from the semi-recumbent position to the strict supine horizontal position, secondary to a passive shift of blood from the splanchnic compartment to the heart, as occurs during passive leg raising [18]; and (2) to transferring the patient from the strict supine to the prone position, an effect that is predominant [17]. On the other hand, the increase in IAP induced by prone positioning also increases the resistance to venous return [17] (Fig. 1). An increase in venous return can be observed when the increase in its pressure gradient is not countered by the increase in its resistance [17] (Fig. 1). This increase in venous return results in an increase in right ventricular (RV) preload, as assessed by the increase in CVP [11, 12, 17]. The increase in CVP may be related to the simple transmission of IAP to the thorax, but this is unlikely as the esophageal pressure increases to a lesser extent than does CVP when patients are transferred from supine to prone position [11, 19].

### Prone Positioning May Decrease Pulmonary Vascular

### Resistance and Right Ventricular Afterload

**Fig. 2:**
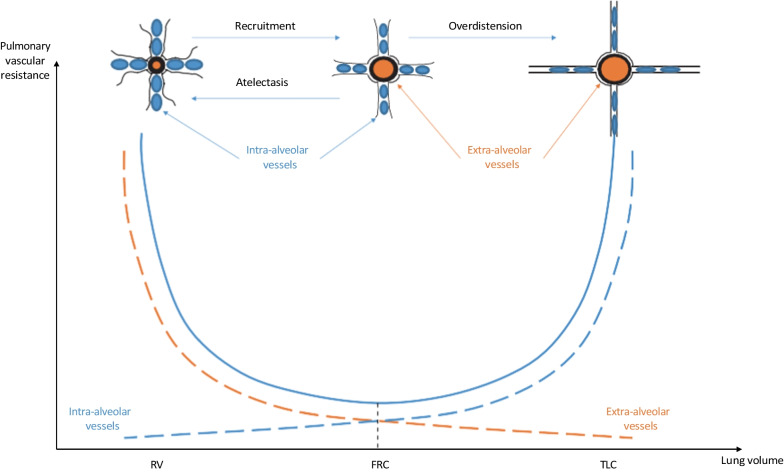
Relationship between lung volume and pulmonary vascular resistance. *FRC* functional residual capacity, *RV* residual volume, *TLC* total lung capacity

In patients with ARDS, RV dysfunction is not rare, its prevalence ranging from 10 to 30% in large observational studies [20–23]. Severe RV dysfunction was shown to be associated with increased mortality [20]. The main cause of RV dysfunction in ARDS is the increase in RV afterload secondary to the increase in pulmonary vascular resistance (PVR). The latter may be due to hypoxic pulmonary vasoconstriction [24], to inflammatory mediators [25], to microthrombi formation [26], and/or to the hemodynamic effects of mechanical ventilation [27, 28]. Regarding the latter mechanism, tidal volume at each insufflation and application of positive end-expiratory pressure (PEEP) over the entire ventilator cycle increase the lung volume. This may increase PVR by compressing the intra-alveolar vessels and, thus, increase the proportion of lung West zones 2, where the alveolar pressure is higher than the pulmonary venous pressure [29] (Fig. 2).

Prone positioning may reverse some of the mechanisms responsible for RV dysfunction during ARDS. First, by improving arterial oxygenation, prone position should decrease hypoxic vasoconstriction [3, 5, 12, 30]. Second, prone positioning allows the recruitment of the vertebral parts of the lungs, resulting in a more homogeneous alveoli aeration [31, 32]. This recruitment of non-aerated alveoli increases the diameter of the extra-alveolar vessels in these regions (Fig. 2). Prone positioning also dampens the overdistension present in hyperinflated lung areas. In this way, it should decrease the compression of intra-alveolar vessels in these zones and decrease the transpulmonary driving pressure, according to principles described by Whittenberger et al. [33] (Fig. 2). Moreover, by recruiting lung units, prone positioning can prevent the application of too high PEEP levels. Finally, prone positioning can increase venous return and central blood volume [17] which, in turn, increases pulmonary venous pressure, releases the compression of some previously compressed pulmonary microvessels, and shifts these vessels from West zone 2 to West zone 3 conditions. The combination of all these effects should result in a decrease in PVR and RV afterload [12, 13] (Fig. 1). It remains to be determined whether the effect of prone positioning on hemodynamics in general and on RV function in particular contributes to its beneficial effect on outcome. In this regard, we have already learned that prone positioning could be beneficial in responders as well as in non-responders defined by changes in blood gas variables (increase in PaO_2_/FiO_2_ or decrease in the arterial carbon dioxide partial pressure (PaCO_2_)) [5, 34].

### Prone Positioning May Increase Left Ventricular Preload

Left ventricular (LV) preload can increase secondary to the improvement in RV preload and afterload with prone positioning, as illustrated by the increase in the pulmonary artery occlusion pressure (PAOP) reported in previous studies [12, 35, 36]. Moreover, in patients with RV enlargement prior to prone positioning, reduction in RV afterload dampens the RV/LV interdependence, as illustrated by the decrease in the RV end-diastolic area/LV end-diastolic area ratio measured by echocardiography. This should also increase LV end-diastolic volume and thus LV preload [12, 13].

### Overall Effects of Prone Positioning on Cardiac Output

Several studies have evaluated the effects of prone positioning on cardiac output with variable results. Some have found that prone positioning has little or no effect [37–39], whereas others have described an increase in cardiac output [13, 40, 41]. In a recent study evaluating 197 prone position sessions in 107 patients, Ruste et al. found that cardiac output decreased ≥15% in 23% of the sessions, increased ≥15% in 25% of the sessions, and remained stable in 52% of the sessions [42]. The changes in cardiac output with prone positioning likely depend on the volume status and the degree of preload responsiveness prior to prone positioning. Indeed, cardiac output increases only in patients with LV preload reserve [12, 17] (Fig. 1). However, cardiac output may decrease in both preload responsive and unresponsive patients if IAP increases to a very large extent with prone positioning, irrespective of the IAP value in the supine position [17]. It is noteworthy that prone positioning can increase venous return, and thus cardiac output, only if the IAP is lower than the intramural pressure of the inferior vena cava (i.e., when the vena cava is in a Takata zone 3) [43, 44]. When profound hypovolemia is present and/or when the IAP under prone position is high, the venous return should be reduced, due to the extension of Takata zone non-3 conditions and to the occurrence of vascular waterfall phenomena [17, 43, 44].

Thus, a major goal when using prone positioning in patients with ARDS should be to minimize the increase in IAP, because a large increase in IAP, independent of its baseline value, may exert some detrimental effects on the circulation [17]. In this regard, in a crossover study, Michelet et al. found that using an air-cushioned mattress was associated with a limited increase in IAP (+ 4 mmHg) compared to a conventional foam mattress (+ 8 mmHg) [36]. Also, the use of thoraco-pelvic supports allows free abdominal movement and may decrease IAP. In a randomized study in 11 patients comparing prone position with or without thoraco-pelvic supports, Chiumello et al. demonstrated that application of thoraco-pelvic supports decreased chest wall compliance, increased pleural pressure, and slightly worsened hemodynamics without any advantage on gas exchange [45]. Moreover, prolonged contact with the supports could induce pressure sores. Finally, the increase in IAP with prone positioning could impair the perfusion of intra-abdominal organs. However, in studies measuring IAP during prone positioning, the increase in IAP was limited and, as long as cardiac output was preserved, hepato splanchnic perfusion [39], liver function [40], gastric intramucosal energy balance [39, 40], and renal function [41] were not impaired. Therefore, the increase in IAP with prone positioning is rather limited. The use of thoraco-pelvic support should be avoided and limited to patients with a high increase in IAP with prone positioning [46].

### Detecting Preload Responsiveness in Patients in the Prone Position

Acute circulatory failure is common in patients with ARDS [8, 47] whether it is secondary to sepsis, vasoplegia induced by sedative drugs, or the side-effects of mechanical ventilation. In the context of ARDS, the optimization of hemodynamics and cardiac output is important since insufficient cardiac output may worsen hypoxemia due to intrapulmonary shunt (low mixed venous oxygen tension effect). Fluid therapy is often considered as the first-line therapy to improve cardiac output and microvasculatory blood flow. However, only one half of patients would effectively benefit from fluid administration in terms of increase in cardiac output, which defines preload responsiveness [48]. Moreover, the pathophysiology of ARDS is marked by increased pulmonary microvascular permeability, with the risk of worsening lung edema when fluids are given. It is also now well demonstrated that the cumulative fluid balance is independently associated with mortality in patients with ARDS [49, 50]. The benefit/risk ratio of fluid therapy should thus be carefully evaluated before any fluid administration, and it is therefore essential to assess preload responsiveness. In this regard, several dynamic indices or tests can be performed [51]. We will review those which have been evaluated and can be used in the prone position during ARDS.

### Trendelenburg Maneuver

The passive leg raising (PLR) test, consisting of transferring a patient from a semi-recumbent position to a position where the trunk is horizontal and the lower limbs are elevated at 30–45° [52], enables the prediction of fluid responsiveness with good reliability [53, 54]. Unfortunately, this postural maneuver is not applicable in the prone position. A Trendelenburg maneuver has been proposed as an alternative, as it mobilizes the blood from the splanchnic venous reservoir and the lower limbs to the intrathoracic compartment, increasing cardiac preload in patients in prone position [55] and receiving extracorporeal membrane oxygenation (ECMO) [56]. In a study of 33 patients with ARDS in the prone position, Yonis et al. found that the area under the receiver operating characteristic curve (AUC) of cardiac output changes during the Trendelenburg maneuver was 0.90 (95% CI 0.80–1.00). An increase in cardiac output ≥8% during the Trendelenburg maneuver enabled the diagnosis of fluid responsiveness with a sensitivity of 87% (95% CI, 67-100), and specificity of 89% (95% CI, 72-100) [55].

### End-Expiratory Occlusion Test

The end-expiratory occlusion test (EEOT) is based on heart-lung interactions. A 15-s expiratory hold transitorily decreases the intrathoracic pressure and thus increases cardiac preload. This increases cardiac output in case of preload responsiveness [57]. An increase in cardiac output ≥5% during an EEOT has been shown to reliably predict fluid responsiveness in patients in the supine position [57, 58]. In the surgical setting, Messina et al. evaluated the performance of an EEOT at 6 and 8 ml/kg to predict fluid responsiveness in 40 patients having spinal surgery in the prone position [59]. They found that an EEOT at either tidal volume value did not reliably predict fluid responsiveness. However, it is important to note that the ventilator interruption during expiration lasted only 15 s, whereas the cardiac output monitor used in that study averaged hemodynamic data over a 30-s period, with a probable dampening of the changes in cardiac output potentially produced by the EEOT [59]. In patients with ARDS, the study by Yonis et al. also found that an EEOT performed with a tidal volume of 6 ml/kg did not predict fluid responsiveness [55]. This may be due to attenuated effects of expiratory hold on venous return increase, since the IAP increase with prone positioning may promote Takata zone 2 conditions in some patients. It must be noted that cardiac arrhythmias were present in 42% of patients in this study [55]. In another study evaluating 84 prone position sessions in patients with severe ARDS with no cardiac arrhythmias, an EEOT at 6 ml/kg was reliable to predict fluid responsiveness with an AUC of 0.93 ± 0.06 (0.87–0.98) but with a low cut-off percent cardiac output increase (3.2%) [60]. Such a low cut-off, which has been reported by other studies [61], requires the use of precise cardiac output monitoring.

### Pulse Pressure Variation

In the absence of cardiac output monitoring, other hemodynamic variables could be used to predict fluid responsiveness. Changes in arterial pulse pressure during mechanical ventilation—also called pulse pressure variation (PPV)—which are secondary to the changes in stroke volume occurring during the respiratory cycle [62], predict fluid responsiveness in patients receiving a tidal volume of at least 8 ml/kg provided they are perfectly adapted to their ventilator and they have no cardiac arrhythmia [63, 64]. Studies evaluating PPV in the operating room setting showed that PPV could predict fluid responsiveness in the prone position [65–67]. Nevertheless, tidal volume was ≥ 8 ml/kg and lung compliance was not impaired in these patients [65–67]. Such results cannot be extrapolated to patients with ARDS, who have low lung compliance and are generally ventilated with low tidal volumes, two conditions that alter the ability of PPV to predict fluid responsiveness [68–70]. In a study evaluating the predictive performance of PPV during prone positioning in patients with ARDS ventilated with a tidal volume of 6 ml/kg, Shi et al. found that PPV ≥ 6.5% enabled preload responsiveness to be assessed with an AUC of 0.85 ± 0.05 (0.77–0.92), a sensitivity of 74% (57%–95%), and a specificity of 79% (56%–96%). However, the gray zone of PPV (between 5 and 8%) included 40% of the cases [60].

### Tidal Volume Challenge

To overcome the limitations of PPV interpretation in case of low tidal volume ventilation, Myatra et al. described a tidal volume challenge (TVC) that consists of a 1-min increase in tidal volume from 6 to 8 ml/kg of predicted body weight [71]. An absolute increase in PPV ≥ 3.5% during a TVC predicted fluid responsiveness in critically ill patients in the supine position [71]. In patients with ARDS undergoing prone positioning, Shi et al. showed that an absolute change in PPV ≥ 3.5% during a TVC assessed preload responsiveness with an AUC of 0.94 ± 0.03 (sensitivity: 98%, specificity: 86%) [60]. The ability of a TVC to predict preload responsiveness was better than that of baseline PPV, but comparable with an EEOT performed at 6 ml/kg [60]. In the study by Yonis et al., the Trendelenburg maneuver performed better than the TVC, but the assessment was performed in only 19 patients, since patients with arrhythmia were excluded [55]. In summary, the TVC, which has been repeatedly found to be reliable in supine patients seems also to be reliable during prone positioning [59, 60]. It has the advantage of assessing preload responsiveness without the need for cardiac output measurements and could be easily used in low and middle-resource settings.

### Mini-Fluid Challenge

The mini-fluid challenge consists of administering a bolus of a small fluid volume (100–150 ml in 1 min) and measuring the changes in hemodynamic variables. The increase in cardiac output after the mini-fluid challenge can predict the increase in cardiac output when administering 400 ml of fluids, for a total of 500 ml fluid administration [72]. This test was shown to be reliable to predict fluid responsiveness in supine conditions [58, 69]. The change in PPV with a mini-fluid challenge could also predict fluid responsiveness [73]. Although it has not yet been evaluated in patients in the prone position, there is no reason why it could not be applicable to these patients. However, in contrast to other above-mentioned tests, the mini-fluid challenge is not reversible as it requires fluid administration.

## Conclusion

Placing a patient with ARDS in the prone position has important implications for both venous return and RV function. While an increase in IAP tends to raise the upstream pressure for venous return, an increase in venous return may be observed only in the absence of a simultaneous rise in the resistance to venous return. Prone positioning can decrease RV afterload, an effect which is beneficial in patients with prior RV dysfunction. On the other hand, prone positioning could reduce cardiac output and organ perfusion in some conditions. Therefore, hemodynamic assessment, including echocardiography and cardiac output monitoring, is important when placing a patient in the prone position. Hemodynamic assessment can also guide fluid management using dynamic tests of preload responsiveness, such as the Trendelenburg maneuver, an EEOT, or a TVC.

## Data Availability

Not applicable.
